# Investigation of Acute Pulmonary Deficits Associated with Biomass Fuel Cookstove Emissions in Rural Bangladesh

**DOI:** 10.3390/ijerph14060641

**Published:** 2017-06-15

**Authors:** Danielle N. Medgyesi, Heather A. Holmes, Jeff E. Angermann

**Affiliations:** 1 School of Community Health Sciences, Division of Health Sciences, University of Nevada, Reno, NV 89557, USA; d.medgyesi@rocketmail.com; 2 Atmospheric Sciences Program, Department of Physics, University of Nevada, Reno, NV 89557, USA; hholmes@unr.edu

**Keywords:** biofuel emissions, cookstove, particulate matter, lung function, chronic obstructive pulmonary disease, spirometry, South Asia, J0101

## Abstract

The use of solid biomass fuels in cookstoves has been associated with chronic health impacts that disproportionately affect women worldwide. Solid fuel stoves that use wood, plant matter, and cow dung are commonly used for household cooking in rural Bangladesh. This study investigates the immediate effects of acute elevated cookstove emission exposures on pulmonary function. Pulmonary function was measured with spirometry before and during cooking to assess changes in respiratory function during exposure to cookstove emissions for 15 females ages 18–65. Cookstove emissions were characterized using continuous measurements of particulate matter (PM_2.5_—aerodynamic diameter <2.5 μm) concentrations at a 1 s time resolution for each household. Several case studies were observed where women ≥40 years who had been cooking for ≥25 years suffered from severe pulmonary impairment. Forced expiratory volume in one second over forced vital capacity (FEV1/FVC) was found to moderately decline (*p* = 0.06) during cooking versus non-cooking in the study cohort. The study found a significant (α < 0.05) negative association between 3- and 10-min maximum PM_2.5_ emissions during cooking and lung function measurements of forced vital capacity (FVC), forced expiratory volume in one second (FEV1), and FEV1/FVC obtained during cooking intervals. This study found that exposure to biomass burning emissions from solid fuel stoves- associated with acute elevated PM_2.5_ concentrations- leads to a decrease in pulmonary function, although further research is needed to ascertain the prolonged (e.g., daily, for multiple years) impacts of acute PM_2.5_ exposure on immediate and sustained respiratory impairment.

## 1. Introduction

The burning of biomass fuels including wood, charcoal, and animal dung in open fire stoves results in incomplete combustion, leading to emissions of particulate matter (PM), carbon monoxide, hydrocarbons, oxygenated organics, free radicals, and chlorinated organics [1]. Organic carbon makes up approximately 50% of all fine particulate mass emitted from biomass burning in cookstoves [2]. Worldwide, it is estimated that 50% of households and 90% of rural households use biomass fuel and coal as a major source of energy [3]. Open fire cookstoves using biofuels have shown to emit as much as 73% more PM_2.5_ (particulate matter with aerodynamic diameter <2.5 μm) than improved stoves incorporating a ventilation system [4]. Thus, the burning of biomass fuels significantly contributes to elevated indoor air pollution (IAP) concentrations.

IAP associated with cookstoves has been linked to several health complications including low birth weight, cardiovascular disease, tuberculosis, cataracts, and other respiratory complications [5]. According to the World Health Organization, stroke, ischemic heart disease, and chronic obstructive pulmonary disorder (COPD) account for more than 80% of the 4.3 million IAP related deaths per year [6]. Of the 2.7 million deaths per year attributable to COPD, 700,000 deaths (over one third) are related to IAP exposure [6]. A survey of households utilizing open fire cookstoves and burning solid fuels in India found about one third of all adults and half of all children experienced symptoms of respiratory illness in the past 30 days [7].

IAP disproportionately affects women and children, as they typically spend more time in the home and cooking meals [8]. Women in rural environments tend to spend several hours preparing and cooking meals for their families. For example, in rural Mexico, women spend 2 to 4 h a day in close proximity to the stove preparing tortillas [9]. Thus, women and the children they care for have an increased risk for respiratory illnesses. For example, acute respiratory infection (ARI) prevalence due to wood and charcoal stoves has been found to be as high as 32% for women and 64% for children in Sierra Leone [10].

Pulmonary function tests, such as spirometry, are commonly used to assess functional aspects of pulmonary health. A study in India found a significant decline in air flow limitation based on forced expiratory volume in one second (FEV1) in women cooking with biomass fuels compared to women utilizing cleaner fuels for cooking [11]. Evaluation of respiratory function between subgroups of women exposed to biomass fuels revealed that animal dung is associated with reductions in FEV1 over forced vital capacity (FEV1/FVC), an indicator of airflow limitation used in the diagnosis of COPD [12,13]. Compared to women using gas stoves, those who use biomass fuels self-reported more phlegm production and were found to have reduced FEV1/FVC, 89.9% vs. 79.9% respectively [14]. Clark et al. [4] found that women self-reported fewer respiratory symptoms such as coughing, phlegm, and wheezing when switching to improved stoves.

The residents of the Naria subdistrict in rural Bangladesh primarily use animal dung, plant matter, and wood for cookstove fuel. Similar biomass fuels were collected from the Dhaka marketplace and used for characterization of organic aerosols in a previous study [2]. Since animal dung and plant matter are prominent sources of low quality fuel (i.e., high polluting) [15], it is crucial to examine health impacts of the use of these rudimentary fuels in order to promote healthier alternatives. Previous studies have established that respiratory illnesses are associated with household air pollution, but this proposed study extends knowledge of this association by (1) assessing the possibility of real- time, ‘rapid-onset’ pulmonary deficits during exposure to biomass smoke; (2) evaluating pulmonary deficits in relation to exposure metrics of particulate matter concentrations via real-time aerosol monitoring; and (3) quantifying the increased magnitude of PM_2.5_ concentrations during cooking after adjusting for baseline ambient concentrations.

## 2. Materials and Methods

Recruitment was conducted through word of mouth and inviting random households throughout the adjacent villages to participate in the study. Women ages 18–65 who stated they were primarily responsible for cooking for the household (inclusion criteria) were invited to participate in the study. Our team intended to recruit women from the general population living in the Naria community, with the awareness that enrolled participants may have pre-existing respiratory illnesses, but due to the lack of access to medical care, many of these cases would be undiagnosed. With the exception of participant 8 (who had sought medical care in Dhaka and was diagnosed with asthma) no other participant had been formally diagnosed with respiratory illnesses, although many complained of chronic respiratory symptoms. Participants received information about the study and provided written consent. As part of the consent process, participants were told why the study was being conducted, what they would be asked to do if they chose to participate, and that they could terminate their participation at any time.

For each household, a survey was conducted by the fieldwork team with the assistance of a Bengali translator. The Bengali translators studied English at a college level and were trained by Duwell International [16]. Surveys were administered in both English and Bengali even though most residents were illiterate in Bengali. Therefore, surveys were read to participants and their responses were recorded in Bengali by the translator and simultaneously in English by a field team member. The survey included information such as family demographics, education, cooking habits, pulmonary health, overall health, and smoking habits to account for confounding factors. Participants were asked to numerically rank the frequency of pulmonary symptoms such as dyspnea, coughing, sputum, and wheezing, from ‘never’ to ‘very often’. Cooking habits were evaluated to determine fuel type, oil type, frequency of cooking meat, and whether participants attempted to avoid smoke. Other questions evaluated the amount of time spent outdoors and whether participants believed there to be substances in the environment that were harmful.

The study was conducted in accordance with the requirements of the Code of Federal Regulations and the Protection of Human Subjects (45 CFR 46 and 21 CFR 50 ad 56). The protocol was reviewed and approved by the University of Nevada Reno Institutional Review Board (IRB #653688) and the Bangladesh Medical Research Council National Research Ethic Committee (Ref: BMRC/NREC/2015-2018/186).

### 2.1. Spirometry

Lung function was measured using the Pony FX Spirometer (Cosmed USA, Chicago, IL, USA). Specifically, lung function measurements of FEV1, FVC, FEV1 over FVC ratio (“Tiffeneau-Pinelli Index”), and peak expiratory flow (PEF) were recorded. In consideration of the cultural norms of the community, participant spirometry measurements were obtained by a single trained female field member, with the assistance of a translator. The field member sat facing the participant and coached participants on the use of the spirometer by demonstrating proper pulmonary function testing technique using a spare disposable expiratory tube. Following a training bout to ensure reproducibility of measurements, participants were first tested while not cooking (>2 h post cooking), and then tested a second time while cooking (30–60 min of smoke exposure). For each spirometry period (non-cooking and cooking), the three most reproducible spirometry bouts were recorded and used for analysis (i.e., the average of the three reproducible bouts for each participant was used for analysis). Reproducible and acceptable tests were determined by measurements of FEV1 and FVC within 5% (or 150 mL) of the two largest maneuvers [17,18]. Participants were (1) tested approximately 2–3 h after cessation of cooking to obtain baseline measurements and (2) tested a second time about 30–60 min into smoke exposure while cooking. By first testing participants before cooking, we were able to account for bias, since spirometry performance is more likely to improve with practice [19]. Improvement in lung function while exposed to smoke may be attributed to improvement with spirometry training.

An a priori power analysis (G*Power v3.1) was conducted which indicated a projected nominal sample size of 54 subjects to discern a statistically significant effect of biomass exposure on FEV1/FVC [4,11,12]. Due to time constraints, cultural, and language barriers, the efficiency and confidence of the pilot study was decreased, restricting the sample size to 15 participants, with only 12 participants having reasonably acceptable spirometry readings and sufficient smoke exposure. Therefore, significant (α ≤ 0.05) cooking pulmonary function changes at the study cohort level were not observed, although a moderately significant decline in FEV1/FVC during cooking was determined (*p* = 0.06). Analysis of decrements of FEV1/FVC, FEV1, and FVC during cooking versus non-cooking were performed using a paired one-sided *t*-test. The average of the three repeated measurements for each participant before cooking and during cooking were calculated and then all qualifying paired measurements (*n* = 12) were included in the analysis. Proportion of predicted FVC and FEV1 measurements used for indoor air pollution (IAP) regression analysis were calculated using the average of the three repeated pulmonary measurements obtained during cooking. Proportion of predicted FVC and FEV1 adjusted for age, height, gender, and ethnicity [20], allowing for inter-participant pulmonary function comparisons with respect to PM_2.5_ exposure. Ethnicity was selected as ‘other’ since the formulas calculated excluded the Indian subcontinent due to variation between data sets collected and analyzed by Quanjer et al. [20].

### 2.2. Air Quality Measurements

For each participant household, a DustTrak 8520 aerosol monitor (TSI Inc., Shoreview, MN, USA) was placed near the cookstove while not in use and again during the burning of biomass fuels (during the afternoon cooking period) to obtain baseline and cooking IAP measurements. All but one participant (who stated she cooked three times a day) reported cooking exclusively during the morning and afternoon, with the afternoon cooking session being the longest. Participants reported the daily time spent cooking each day ranged from 1.5 to 4.5 h, with the average being 2.5 h, and the mode (7 participants) reporting cooking 3 h per day. Therefore, cooking habits across the community appear to be similar, allowing for emission exposure comparisons between participants. Aerosol monitors were placed on a stool to maintain consistent height between households and while effort was made to also keep the proximity to the stove consistent, this was not always possible. Therefore, the distance of the monitors from the stove was measured to account for variability in the data from different homes. The DustTrak monitors were programmed to sample PM_2.5_ mass concentrations at a one second time resolution. Additionally, fuel type, shelter ventilation, and movement near the stove were noted by the fieldwork team during the sampling.

Filter based PM_2.5_ mass concentration measurements were not available to calibrate the DustTrak data for aerosol from biomass combustion. Since the DustTrak monitors are an aerosol optical counter and the mass concentrations from the instrument are calibrated for Arizona Dust [21] the mass concentrations measured in this study cannot be used in a quantitative manner. Therefore, air quality measurements presented in this work are normalized to determine the IAP during cooking time periods.

Indoor air pollution metrics (IAP_cooking_, in the equation below) were calculated as a function of baseline and ambient concentrations to minimize bias associated with using two aerosol monitors (allowing IAP in two homes to be monitored simultaneously) and allow for comparison between all homes in the study. Post processing steps were performed to average the 1-s data and estimate exposure metrics for cooking. Specifically, 3- and 10-min maximum values were obtained by calculating a running mean of the 1-s data collected during cooking using 3- and 10-min sliding window averages. The maximum average for the respective running means were selected and used as the maximum value, or exposure metric for IAP associated with cooking. The 10-min baseline average was acquired using a 10-min sliding average for the 1-s data collected during non-cooking, then a total average was calculated from the sliding averages. To normalize the air quality data from each home, individual exposure metrics were calculated as the 3-min maximum and 10-min maximum PM_2.5_ (μg/m^3^) minus 10-min average baseline. An additional normalization step was used to present the exposure metrics as a function of ambient concentrations. To do this, the exposure metrics are expressed as a multiplicative increase of the average baseline concentrations (10-min non-cooking averages) from all 15 homes (i.e., ambient concentration):

(1) $$IAP_{cooking}=\frac{3\;(\mathrm{or}\;10)\;\min\;\max\;PM_{2.5}\;\mathrm{during}\;\mathrm{cooking}\;(\mathrm{individ}.\mathrm{home})-10\;\min\;\mathrm{avg}\mathrm{baseline}\;PM_{2.5},\;\mathrm{no}\;\mathrm{cooking}\;(\mathrm{individ}.\mathrm{home})}{\text{average of all households 10 min avg baseline }PM_{2.5}\;(\mathrm{ambient})}$$

## 3. Results

As shown in Table 1, the study acquired complete spirometry, air quality and survey data for 15 participants. Of those 15 participants, one participant was under the age of 20, six were between the ages of 21–39, and eight were over the age of 40. Three participants had been cooking for less than a year, six participants had been cooking for 10–25 years, and six participants had been cooking for more than 25 years. 60% of participants utilized a combination of all three biomass fuel types (cow dung, plant matter, and wood), 80% of all participants utilized cow dung, and 20% utilized wood only. Plant matter was defined as any combination of rice straw, dry leaves, crop residue, and twigs. 

**Figure 1:**
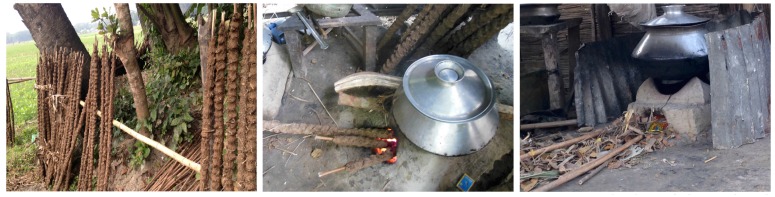
Typical cookstove fuels in rural Bangladesh: (**left**) Cow dung sticks, (**middle**) Stove burning cow dung, and (**right**) Stove burning plant matter.

Additionally, waste such as paper and plastic wrappers were observed to be mixed into the plant matter and used as cookstove fuel. Cow dung was molded onto wooden sticks and set outside to dry; cow dung sticks and other materials such as plant matter were then placed in ground level traditional stoves as shown in Figure 1. Typically, depending on the season and availability of fuel sources, households would use the fuels they could forage. The cooking areas were separate from the main house and primarily constructed from bamboo, straw, burlap cloth, and tin. Ventilation varied from large cooking areas with open ventilation (e.g., minimal wind cover, straw construction) to small areas with limited ventilation (e.g., fully enclosed room with roof, tin construction), and one participant’s home had a chimney on their cookstove. Survey data was completed for 17 participants, but spirometry and air quality data were not obtained for two participants due to cultural barriers and illness. Of the women surveyed, 8 of 17 (47%) attempted to avoid the smoke during cooking, mainly by covering their face with a sari (one participant had a chimney). As shown in Table 2, the most common symptoms encountered during cooking were difficulty breathing (29.4%) and coughing (17.6%). No participants reported smoking tobacco products, but 47% of women (mostly 40 years and older) reported chewing betel leaves.

### 3.1. Air Quality Measurements

**Figure 2:**
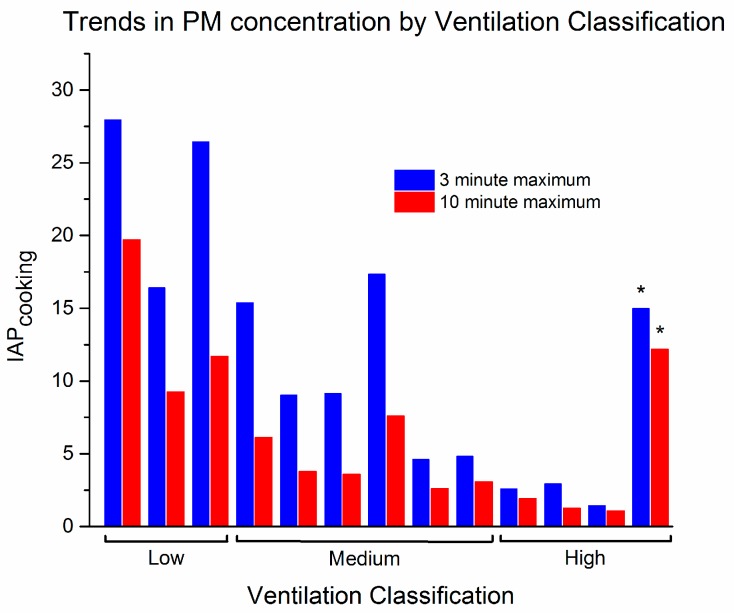
Comparison of ventilation classification and particulate matter concentrations. Groups of homes sorted by ventilation classification were found to have significantly different ambient PM_2.5_ loads by one-way ANOVA (3 min maximum data: Prob>F = 0.0016; 10 min maximum data: Prob>F = 0.0017). * Home 9 with chimney ventilation.

**Figure 3:**
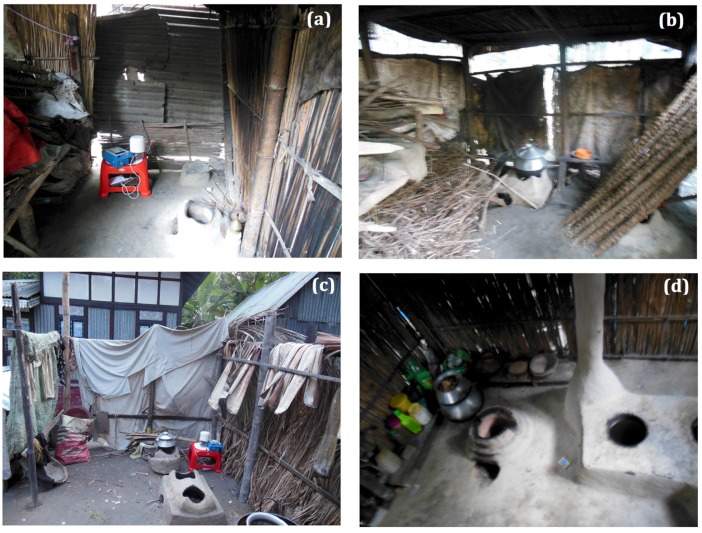
Ventilation classification for homes in rural Bangladesh: (**a**) Low ventilation; (**b**) Medium ventilation; (**c**) High ventilation; and (**d**) Stove with chimney.

Although quantitative measurements cannot be reported for the PM_2.5_ mass concentrations from this study, qualitative comparisons in PM_2.5_ concentrations between cooking and non-cooking time periods and different households can be investigated using the normalized PM_2.5_ metrics. Ventilation appeared to be a primary factor contributing to near-stove PM_2.5_ loads. As shown in Figure 2 ventilation was qualitatively grouped as low, medium, or high based on number of sides covered, presence or absence of roof, and materials used for shelter. The air exchange rate in each kitchen area was not measured and these types of measurements should be considered in future work. Low ventilation was categorized as a small area where airflow was constricted by certain materials such as tin, versus straw or wood, used for shelter walls, see Figure 3a. Medium ventilation was defined as having three sides and a roof, which was observed to be the typical cooking shelter (46%); see Figure 3b. High ventilation was defined when cooking areas contained less sides and used materials that allowed for greater air flow such as straw walls, see Figure 3c. Home 9 was defined as having high ventilation because the stove contained a chimney, see Figure 3d. However, as shown in Figure 2, Home 9 had the highest concentrations of PM_2.5_ compared to other homes with high ventilation. This finding is important because it suggests that when chimneys are not constructed properly they may not increase ventilation, but rather restrict airflow or negatively impact the air/fuel ratio for efficient combustion leading to increased PM_2.5_ concentrations.

**Figure 4:**
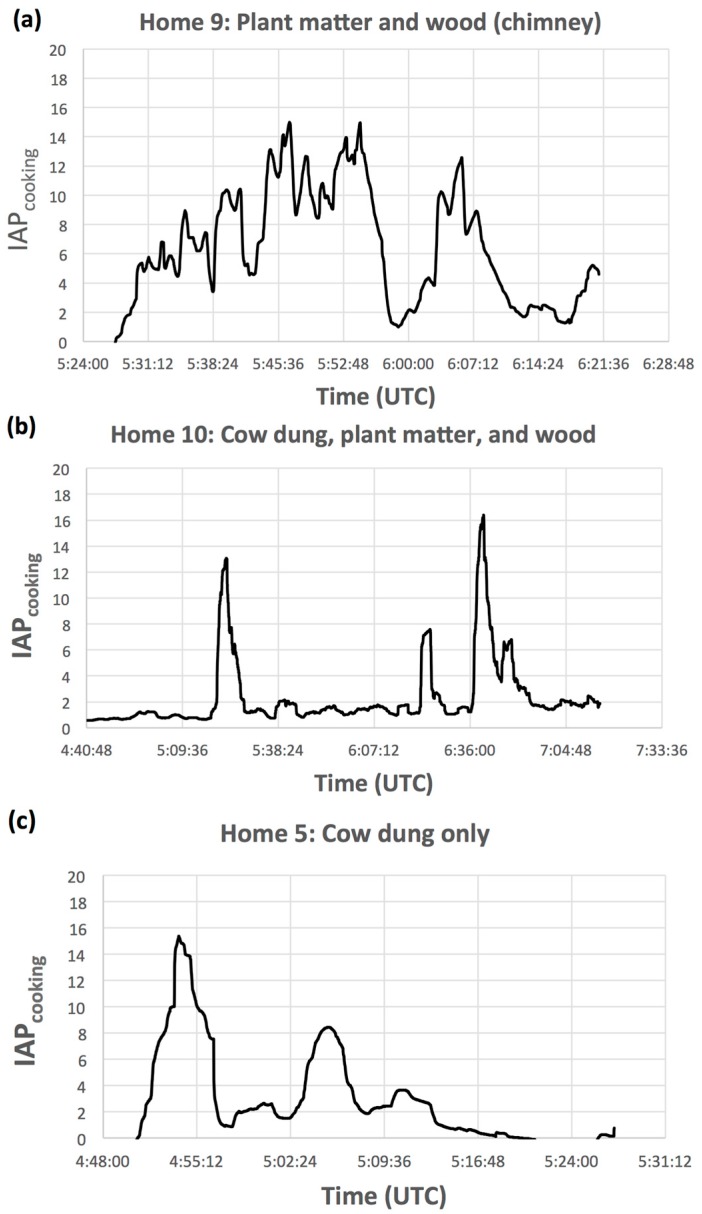
Time series of 3-min average indoor air pollution (IAP) data for each biomass fuel type common in rural Bangladesh: (**a**) Plant matter and wood; (**b**) Cow dung, plant matter, and wood; and (**c**) Cow dung only.

As shown in Figure 4a–c time series PM_2.5_ concentration patterns varied depending on the type of biomass fuel used. Acute, elevated spikes in PM_2.5_ were observed when plant matter was burned. Stoves that utilized cow dung were observed to have elevated PM_2.5_, but average instantaneous peak height was lower than plant matter peaks and longer in duration, indicative of the slower-burning nature of this fuel source. The different behavior of the time series PM_2.5_ concentrations for the different biomass fuels is expected because the fuels have different energy densities. The energy density difference leads to different burning characteristics for the fuels, and therefore different emissions factors [22]. Not only are the total emissions for the different fuels expected to be different, the behavior of the emissions factor for each fuel type is also a function of time [23]. Both are indicative of variation in fuel type energy content, consistent with different ‘energy ladder’ rankings—where less energy-dense fuels do not burn as cleanly, and have increased emissions rates. Quantification of IAP emission profile as a function of time is critical in the effort to determine the health impacts associated with cookstove emissions because acute, elevated peaks may be related to harmful exposures that are generally unrepresented in long-term averaged IAP data (e.g., 1-h averages and greater).

### 3.2. Pulmonary Health

Three participants were excluded from the pulmonary health analysis due to insufficient smoke exposure (1 and 9B) and unsatisfactory technique during the first spirometry bout (15). Twelve qualifying participants were included in the pulmonary health analysis. Significant non-cooking and cooking pulmonary function changes were not observed, although a moderately significant decline in FEV1/FVC at the study cohort level during cooking, compared to the paired non-cooking spirometry bout was observed (*p* = 0.06). Decline in FEV1/FVC during cooking coincides with previous findings of FEV1/FVC decline in women using dung for biomass fuel [12], and is indicative of airflow limitation, used in the diagnosis of COPD [13]. Decline in FEV1 and FVC during cooking were non-significant (*p* = 0.15 and *p* = 0.26, respectively). Factors that attributed to non-significance include but are not limited to: small sample size, time constraints, cultural and language barriers, participants’ unfamiliarity with spirometry, inaccuracy in using spirometry, and improvement during the second bout of spirometry testing while cooking. However, as shown in Table 1, over a third (42%) of qualifying participants displayed decrements in FEV1 greater than 5% following acute smoke exposure, with participant 2 have the most severe decrement (91%). The Pony FX Spirometer diagnosed two participants (8 and 14) as having ‘severe impairment’ (FEV1 < 50% predicted) both while not cooking and during cooking. One participant (2) displayed a major decrement in lung function while cooking and was therefore diagnosed as having very severe impairment (FEV1 < 30% predicted) during her second test. All three women who suffered from ‘severe to very severe impairment’ were 40 years and older and had been cooking for more than 25 years.

**Figure 5:**
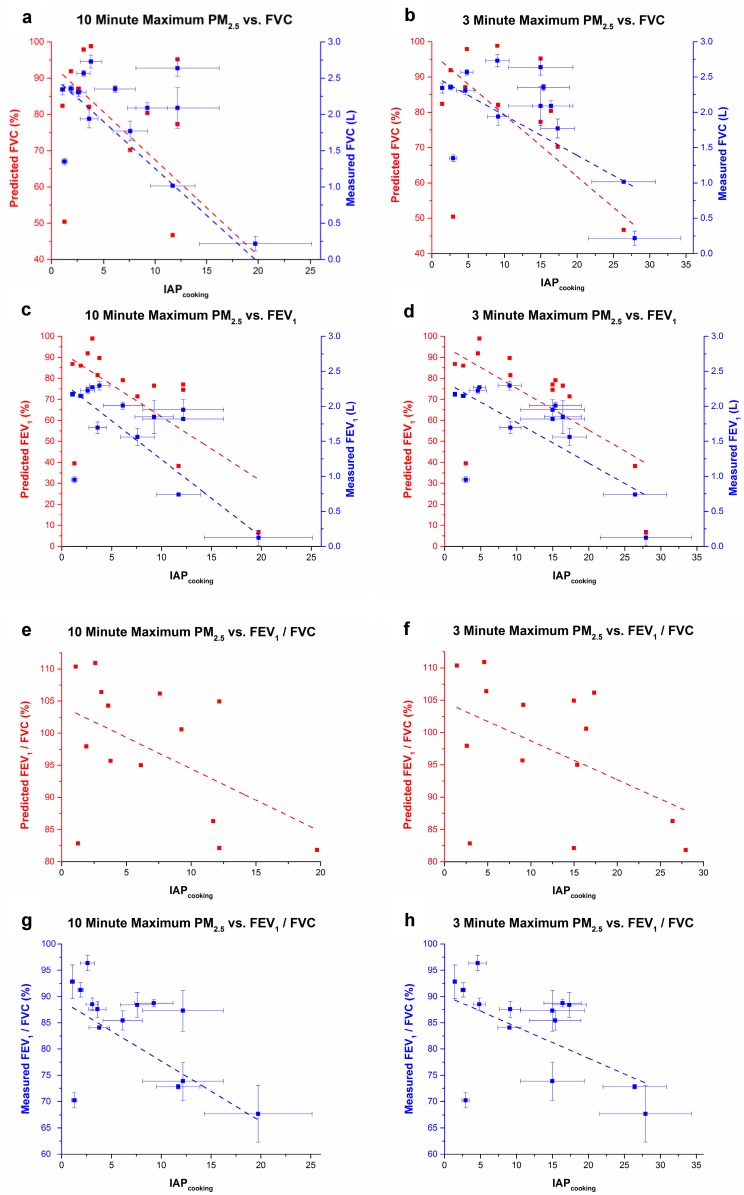
(**a**–**h**). Linear fits and regressions of PM_2.5_ levels against measured and % predicted Pulmonary Function Test parameters. Whisker bars indicate standard deviations for measured FEV and FVC data. ANOVA analyses of linear fits are summarized in Table 3 (**a**–**h**).

As indicated in Figure 5, air quality measurements were compared to average lung function values measured for each qualifying participant (*n* = 11) using linear regression to quantify the relationship between increased IAP_cooking_ and decreased lung function [24]. Participant 4 was excluded from the linear regression due to discrepancies between recorded PM_2.5_ data and cooking observations noted by the field research team. For this home, PM_2.5_ concentrations were about 74 and 29 times the ambient for the 3-min and 10-min maximum, respectively, which is not realistic based on visual observations, therefore it is likely that the instrument was tampered with during sampling. PM_2.5_ concentrations during cooking in all homes included in the regression ranged from 2 to 29 times ambient levels and 2 to 21 times ambient levels for 3- and 10-min maxima, respectively. IAP_cooking_ values that were further adjusted for background PM_2.5_ levels and used in the regression ranged from 1.4 to 27.9 for 3-min maximum and 1.1 to 19.7 for 10-min maximum. For comparison, measured IAP_cooking_ loads were also regressed against observed lung function measurements expressed as a percent of predicted pulmonary function parameters, adjusted for age, height, gender, and ethnicity [20]. 3- and 10-min maximum IAP_cooking_ measurements were significantly associated (α < 0.05) with instantaneous decrements in average and percent of predicted values of FEV1 and FVC (Table 3). Effect estimates for the ‘measured model’ regressions forecast decrements of 60 to 130 mL FVC, 50 to 100 mL FEV1, and 0.6 to 1.15% FEV1/FVC for each unitary increase in IAP_cooking_. Effect estimates for the ‘predicted model’ regressions forecast decrements of 1.8 to 2.6% FVC, 2.0 to 3.1% FEV1, and 0.6–1.0% FEV1/FVC for each unitary increase in IAP_cooking_. These findings demonstrate that changes in pulmonary function are likely occurring due to acute high PM_2.5_ exposure to biomass fuel emissions.

## 4. Discussion

Our study found a moderately significant FEV1/FVC decline during cooking (*p* = 0.06) when compared to paired non-cooking spirometry bouts in the study cohort, despite an insufficient sample size. This is consistent with previous studies that have found an association between the use of biomass fuels used for cooking and a decline in FEV1/FVC function [12,14], although this is the first study to our knowledge that has evaluated instantaneous declines in FEV1/FVC during biomass fuel cookstove exposure in a rural region located in a developing country.

Over one third of our study cohort complained of respiratory symptoms (e.g., cough, phlegm, difficulty breathing) during cooking, consistent with another study conducted in India [7]. The daily amount of time spent cooking, or exposure time period of solid fuel stove emissions, in our study was similar to daily cooking durations reported in other studies. For example, a cohort of women in rural Mexico reported cooking 2–4 h a day [9], which is reflective of our study cohort’s reported average cooking time of 2.5 h. Although our study did not find a significant decline in FEV1 while cooking when compared to non-cooking (*p* = 0.15), a significant negative association between IAP_cooking_ and FEV1 during cooking is reflective of Bihari et al.’s findings of diminished FEV1 function associated with biomass burning emissions [11].

The Global Initiative for Chronic Obstructive Lung Disease (GOLD) standard of FEV1/FVC < 0.70 for obstructive disorders has been implemented as an epidemiological principle by the Burden of Obstructive Lung Disease (BOLD) initiative and the Latin-American Project for the Investigation of Pulmonary Obstruction (PLATINO) [25]. Participants 2 and 8 had an FEV1/FVC ratio at or less than 0.70 (0.68 and 0.70, respectively) and participant 14 had an FEV1/FVC of 0.73. In addition, all three participants had a predicted FEV1 of less than 0.50, demonstrating GOLD III severe COPD [13]. The combination of higher FEV1/FVC ratios with very low FEV1 predicted values may be explained by consideration of the following two phenomena: FEV1/FVC ratios can be overestimated due to failure of a subset of participants to completely express FVC [25], and very low FEV1 predicted values may be partially explained by the idea that Asian Indian, Polynesian and Mongoloid people have comparatively smaller lung compacities compared to other ethnic groups [26]. Since there has yet to be an accurate prediction model for the Indian subcontinent, our prediction values were adjusted for ‘other’ ethnicity [20]. Therefore, prediction models may overestimate FVC and underestimate FEV1, thereby decreasing the ‘percent of predicted’ performance of study participants; this observation may partially explain the discrepancy in significance between measured and predicted regressions of 3- and 10-min-maximum PM_2.5_ vs. FEV1/FVC (Table 3). These issues warrant further attention due to the intensity of IAP exposure on the Indian subcontinent and the importance of the FEV1/FVC ratio as a classifier of COPD status. 

Chronic obstructive pulmonary disorder (COPD) is a persistent disorder in which an individual has dyspnea, chronic cough or sputum production, and a history of exposure to risk factors, such as smoke from home cooking and heating fuels [13]. Although we were not able to diagnose participants as having chronic obstructive disorder, there is evidence that a few participants suffered from chronic pulmonary illness. Three women (2, 8, and 14) in particular had notably depleted lung function. Of the three women, two (8 and 14) displayed ‘severe impairment’ (FEV1 < 50%) both while not cooking and cooking. This is indicative of a chronic problem. We observed another case in which one participant (2) displayed normal spirometry while not cooking, but ‘very severe impairment’ (FEV1 < 30%) while cooking. This particular case was also found to have an extraordinarily enclosed area (i.e., small ceiling height ~1.5 m, fully enclosed, with a roof and a door for entry) with noticeably more smoke where the 3-min maximum PM_2.5_ concentrations during cooking were 27.9 times the ambient concentrations. Based on survey results, all three women complained of persistent pulmonary health complications including difficulty breathing, coughing, and phlegm production.

Since this was a pilot study, there were several factors that decreased the study’s confidence and efficiency. Cultural and language factors became barriers that contributed to a decreased sample size. Researchers quickly found that women who participated in the study did not like to be interrupted while they were tediously preparing meals for their families. Spirometry also posed a challenge for the women, as most had never experienced such a device. Ensuring the women were comfortable and encouraging them to exhale with force was critical in receiving satisfactory results. Performing spirometry in a clinical setting with a female doctor present could increase the comfort level of participants. Unfortunately, the study did not have the means to calibrate the aerosol data due to difficulties with filters and humidity affecting the aerosol monitors. Obstacles identified while conducting the field work can contribute to the modification and improvement of future studies. Future collection of filter-based PM_2.5_ mass measurements to calibrate aerosol data will allow for PM_2.5_ concentrations to be compared quantitatively and related to the World Health Organization standards [27].

## 5. Conclusions

This pilot study found that exposure to biomass burning emissions from solid fuel stoves, associated with acute elevated PM_2.5_ concentrations, leads to a decrease in pulmonary function, although further research is needed to decipher prolonged (daily for multiple years) acute PM_2.5_ exposure impacts on immediate and sustained respiratory impairment. Our study was unable to determine whether pulmonary deficits associated with acute elevated PM_2.5_ are instantaneous, chronic, or a combination of both, which we now believe the prior to be true based on our findings. Older women who had been cooking for more than 25 years displayed signs and complained of symptoms associated with chronic respiratory illness. A few younger participants experienced decrements in pulmonary function only while cooking, illustrating signs of acute pulmonary suppression that could later develop into chronic pulmonary deficits. It would be helpful for future work to decipher physiological impacts of acute elevated PM exposure on those who do and do not display symptoms of chronic respiratory illness.

Significant regression trends between suppressed pulmonary function and increased IAP, and further significant association between increased IAP and ventilation demonstrates the importance of adequate ventilation in areas with cookstoves. Future studies should consider effective and culturally-accepted techniques to increase ventilation and decrease exposure in order to improve quality of life and working conditions for rural Bengali women.

The nonexistent regulation of healthcare providers in Bangladesh will continue to impact the quality of care Bengalis receive. ‘Village doctors’ may practice allopathic and alternative care including faith-healing which can be ineffective and possibly harmful to patients [28]. One woman in this study told of her struggle to seek treatment for her pulmonary illness. She had visited a doctor and was diagnosed with asthma and given a bronchodilator to help relieve the symptoms. Her pulmonary health complications were severe enough that she traveled to Dhaka to seek quality health care, but found no relief for her symptoms. She continues to suffer and does not find the bronchodilator inhaler to be sufficient. Our suspicion is that she was misdiagnosed, and is likely suffering from COPD. It is unfortunate to know the residents of Bangladesh do not have access to the proper healthcare they desperately need. Not to mention, healthcare is tremendously difficult to obtain due to poverty.

The University of Nevada, Reno School of Medicine continues to collaborate with Duwell International to provide the residents of Naria with quality care. Future work such as holding education sessions discussing the health impacts of burning biomass fuels could increase awareness and improve respiratory health outcomes. Although it may not be feasible for the residents to switch to cleaner fuels due to finances, suggesting ways to avoid smoke exposure and increase ventilation would be a step forward. Additionally, it is critical to educate women on the hazards of exposing children to cookstove smoke. We observed several cases in which the women carry babies while cooking in front of the stove. During the field study we also observed that many children 12 months and younger were given cough suppressants to relieve respiratory symptoms. Although mothers complained of their children suffering from colds and even cases of pneumonia, they did not appear to associate their children’s pulmonary illness with cookstove smoke. Future opportunities for medical residents to gain experience abroad can provide women and children suffering from pulmonary illness the proper care that otherwise may not be accessible. Community involvement on a multitude of levels can be mutually beneficial by improving (1) local knowledge of health related issues and (2) the public perception of the research community [29].

## Figures and Tables

**Table 1:** Demographics and characteristics of participating homes.

Demographics					Measured Non-Cooking *** IAP Variability	Measured Cooking IAP Level		Lung Function Decrements				
								While Cooking				
Subj/Home	Age	Years Cooking	Fuels Utilized	Ventilation Class				**** % FEV1/FVC ^†^		FEV1		FEV1% Change
					Baseline Differences between Homes	3′ Max	10′ Max	Non-Cooking	Cooking	Non-Cooking	Cooking	$\left(\frac{Avg_{cook}-Avg_{noncook}}{\begin{array}{c} Avg_{noncook} \end{array}}\right)$ * 100
								Average (Standard Deviation), *n* = 3				
1	18	2–3 days	W	high	−0.02	2.6	1.9	NA	NA	NA	NA	NA
2	60	30	CD, PM, W	low	0.09	27.9	19.7	91.50 (0.42)	67.7 (5.37)	1.39 (0.03)	0.12 (0.11)	−91.11
4	21	4–5 months	CD, PM, W	low	0.09	NA	NA	85.33 (4.16)	83.97 (2.33)	2.02 (0.21)	2.24 (0.10)	11.06
5	25	14	CD, PM, W	medium	0.08	15.4	6.1	88.03 (0.96)	85.43 (1.82)	2.12 (0.11)	2.01 (0.05)	−5.04
6	30	15	CD, W	medium	−0.05	9	3.8	84.87 (3.07)	84.10 (0.30)	2.44 (0.06)	2.30 (0.06)	−6.00
8	40	30	W **	high	0.01	2.9	1.3	70.00 (1.20)	70.23 (1.43)	0.95 (0.02)	0.95 (0.04)	0.35
9A	25	2–3 months	CD, PM, W	high	0.01	15	12.2	86.00 (10.06)	73.87 (3.65)	2.20 (0.32)	1.95 (0.15)	−11.36
9B	50	30	CD, PM, W	high	0.01	15	12.2	NA	NA	NA	NA	NA
10	30	16	CD, PM, W	low	−0.25	16.4	9.3	88.83 (1.40)	88.77 (0.75)	1.98 (0.03)	1.85 (0.24)	−6.41
11	50	38	CD, PM, W	medium	−0.11	9.1	3.6	88.70 (1.47)	87.57 (1.54)	1.57 (0.07)	1.70 (0.08)	8.07
12	45	25	CD, PM, W	high	−0.04	1.4	1.1	91.07 (2.44)	92.83 (3.20)	2.11 (0.02)	2.17 (0.03)	3.16
14	50	34	CD, W *	low	0.04	26.4	11.7	71.13 (1.33)	72.80 (0.44)	0.76 (0.03)	0.74 (0.01)	−2.20
15	55	43	CD, PM, W	medium	0.08	17.3	7.6	NA	NA	NA	NA	NA
16	35	12	CD, PM	medium	0.06	4.6	2.6	99.23 (0.49)	96.40 (1.48)	2.21 (0.03)	2.23 (0.04)	0.75
17	50	25	W	medium	0.00	4.8	3.1	89.43 (0.46)	88.53 (1.19)	2.22 (0.04)	2.27 (0.02)	2.25

^†^ Moderate significant decline during cooking via paired one-sided *t*-test (*p* = 0.06). CD: Cow Dung; PM: Plant Matter; W: Wood; * Fire started with oil; ** Wood chips/shavings; *** Indoor Air Pollution (IAP) Variability calculated as subject baseline 10 min average—Average all baseline 10 min averages.**** Forced expiratory volume in one second over forced vital capacity (FEV1/FVC) expressed as a percent.

**Table 2:** Survey-reported health issues.

General Symptoms Encountered While Cooking		Cardiorespiratory Symptoms	
Symptom	Prevalence (%)	Symptom	Prevalence (%)
difficulty breathing	29	ever chest pain	53
coughing	18	chest pain walking uphill	53
wheezing	12	chest pain walking level	29
headache	12	severe chest pain >30′	29
phlegm	6	shortness of breath	41
watery eyes	6		
*N* = 17			

**Table 3:** ANOVA analyses of linear fit regressions ^†^.

Model	Measurement	Pearson’s R	Adj. R Square	F Value	Prob>F	EE *
Measured	(a) 10 min max PM_2.5_ v FVC	−0.866	0.729	36.00	0.00006	−0.13
% Predicted	(a) 10 min max PM_2.5_ v FVC	−0.605	0.313	6.92	0.01	−2.64
Measured	(b) 3 min max PM_2.5_ v FVC	−0.886	0.767	43.90	0.00002	−0.06
% Predicted	(b) 3 min max PM_2.5_ v FVC	−0.620	0.333	7.49	0.02	−1.75
Measured	(c) 10 min max PM_2.5_ v FEV1	−0.813	0.632	23.34	0.0004	−0.11
% Predicted	(c) 10 min max PM_2.5_ v FEV1	−0.663	0.393	9.17	0.01	−3.06
Measured	(d) 3 min max PM_2.5_ v FEV1	−0.936	0.865	84.48	<0.00001	−0.05
% Predicted	(d) 3 min max PM_2.5_ v FEV1	−0.663	0.393	9.41	0.01	−1.99
Measured	(e) 10 min max PM_2.5_ v FEV1/FVC	−0.667	0.399	9.63	0.009	−1.15
% Predicted	(g) 10 min max PM_2.5_ v FEV1/FVC	−0.510	0.198	4.22	0.06	−0.98
Measured	(f) 3 min max PM_2.5_ v FEV1/FVC	−0.749	0.525	15.36	0.002	−0.60
% Predicted	(h) 3 min max PM_2.5_ v FEV1/FVC	−0.487	0.173	3.72	0.08	−0.60

* EE = Effect Estimate derived from slope factor (percent or mL pulmonary function change per unit increase in IAP_cooking_). ^†^ *n* = 11 for all ANOVA analyses.
